# Resistance to Change as a Mediator Between Conscientiousness and Teachers’ Job Satisfaction. The Moderating Role of Learning Goals Orientation

**DOI:** 10.3389/fpsyg.2021.757681

**Published:** 2022-01-24

**Authors:** Ramona Paloş, Delia Vîrgă, Mariana Craşovan

**Affiliations:** ^1^Department of Psychology, West University of Timişoara, Timişoara, Romania; ^2^Department of Educational Sciences, West University of Timişoara, Timişoara, Romania

**Keywords:** teachers’ job satisfaction, conscientiousness, resistance to change, achievement goals orientation, moderation

## Abstract

Teachers’ job satisfaction has been the subject of many studies that tried to identify its main sources. Based on the social cognitive career theory, the present study aimed to investigate the relationships between personality traits, goals orientation, and teachers’ job satisfaction. A total of 321 Romanian teachers completed an online questionnaire. The results demonstrated new insights regarding the relationships between psychological variables (conscientiousness, dispositional resistance to change, and achievement goals orientation) and teachers’ job satisfaction. Cognitive rigidity, as a mechanism to resistance to change, mediates between conscientiousness and teachers’ job satisfaction. Moreover, the moderation role of learning goals orientation manifests in the relation between conscientiousness and job satisfaction. These findings emphasize that school management needs to offer teachers information and explain the change’s benefits if they want to prevent individual resistance to change and decrease satisfaction related to their work.

## Introduction

Working in a competitive educational environment that challenges them to be efficient and succeed in their job (Butler, 2007; Avidov-Ungar and Magen-Nagar, 2014), teachers are constantly facing change and continually need to improve their competencies (Toropova et al., 2021). If the benefits of these changes are not properly explained, teachers may feel stressed and uncomfortable, developing resistance toward changes (Michel et al., 2013), which may create demotivation and lack of job satisfaction (Evans, 2000; Gori and Topino, 2020).

Job satisfaction, as a general attitudinal evaluation of work (Mount et al., 2006), has been the subject of many studies that tried to identify its main sources (Zembylas and Papanastasiou, 2006; Lent and Brown, 2008; Lent et al., 2011; Skaalvik and Skaalvik, 2013). Consequently, three broad categories of variables were determined: environmental (e.g., the work itself, job role-related characteristics, or working conditions), psychological (e.g., personality aspects, attitudes, and motivation), and demographic (e.g., age, gender, and tenure) (Perrachione et al., 2008; Ghavifekr and Pillai, 2016). Many of these variables were included in different models as predictors for job satisfaction. For example, based on the social cognitive career theory (Lent et al., 1994; Lent and Brown, 2006) built an empirical model that explains the relationships between job satisfaction and (a) “personality and affective traits, (b) goals and goal-directed activity, (c) self-efficacy, (d) work/educational satisfaction, (e) work conditions and outcomes, and (f) goal-relevant environmental supports, resources, and obstacles” (Lent and Brown, 2006, p. 238). Being tested in several educational and cultural environments (Lent et al., 2011; Badri et al., 2013; Perera et al., 2018), results supported the relationships between its variables.

Starting from this model and based on previous evidence, the present study aimed to investigate the relationships between personality traits, goals orientation, and job satisfaction. Thus, conscientiousness and dispositional resistance to change were analyzed as personality traits. Conscientiousness was chosen because of its strong relationship with job satisfaction. Cognitive rigidity, one of the dispositional resistance to change components (Oreg, 2006; Oreg et al., 2008), was introduced as a new element to investigate its impact on teachers’ job satisfaction in a changing world. Because teachers need to develop their competencies to cope with these changes continually, learning goals orientation was examined in relation to job satisfaction.

Conscientiousness, as a personality feature, displayed a strong correlation with job satisfaction (Judge et al., 2002; Lapierre and Hackett, 2007). However, its positive effects have not been widely exploited in the literature (Judge et al., 2002). People with a high level of conscientiousness (e.g., responsible, hard-working, and goal-oriented) are persevering in achieving success (Topino et al., 2021), and are more likely to do the job better than their less conscientious colleagues. This increases their sense of accomplishment and helps them to experience job satisfaction (Mount et al., 2006). Moreover, it seems that job satisfaction is influenced by the joy that people feel when they excel in the things they do, and their performance brings them recognition, and consequently, job satisfaction (Steel et al., 2019).

Considered as an “individual’s personality-based inclination to resist changes” (Oreg, 2018, p. 2), dispositional resistance to change is another personality trait with an impact on teachers’ job satisfaction. Cognitive rigidity (reluctance to consider alternatives), was found to be a significant predictor for teachers’ behavioral resistance (Kalman and Bozbayindir, 2017), with an impact on their emotional responses toward change (Oreg, 2006), and finally, on the job satisfaction. Changes in education put pressure on teachers to change themselves and their practices (Dinham and Scott, 2000). Those of them with a high level of cognitive rigidity are willing to keep their traditional methods and way of teaching if they perceive changes as increasing the workload or if they have the feeling of “the uselessness of their existing skills and competencies” (Kalman and Bozbayindir, 2017, p. 145).

The way the teachers cope with change situations or how they define and strive for success is determined by their personal achievement goals orientation (Parker et al., 2012), representing the motivational aim to be involved in a specific task (Sparfeldt et al., 2015). Although significant research has been developed on teachers’ goals orientation, rather less attention has been paid to their relationship to job satisfaction (Skaalvik and Skaalvik, 2013; Han et al., 2016). However, some of these studies reported positive correlations with learning goals, negative with performance-avoidance goals, and inconsistent relationships (positive or zero) with performance-approach goals (Papaioannou and Christodoulidis, 2007; Skaalvik and Skaalvik, 2013).

**FIGURE 1:**
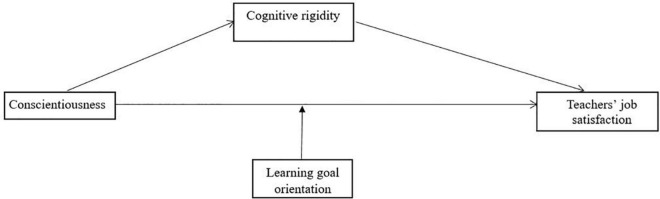
Theoretical model.

Conscientiousness and resistance to change are personality traits that need to be studied more thoroughly (Amarantou et al., 2018), together with achievement motivation, to understand their impact on teachers’ job satisfaction. Consequently, we proposed a parsimonious model with all these three variables as predictors for teachers’ job satisfaction (Figure 1). To test the direct and indirect relationships, we specified a theoretical model in which conscientiousness is related to teachers’ satisfaction both directly and indirectly through resistance to change dimensions (i.e., cognitive rigidity). Also, we tested the moderating role of learning goals orientation in the relation between conscientiousness and job satisfaction.

Teachers’ job satisfaction is an essential component of the educational system because it contributes to teacher retention, commitment, and school effectiveness (Liu and Onwuegbuzie, 2014). Thus, our results will provide supplementary theoretical information contributing to the increase of the Lent and Brown (2006) model’s explanatory value. From the practical perspective, it will emphasize strategies that can be improved to ensure teachers’ job satisfaction when school management implements reforms.

### Conscientiousness and Job Satisfaction

Conscientiousness, one of the five dimensions of the personality, characterizes scrupulous, diligent, persistent, and well-organized persons (Costa and McCrae, 1992). People with a high level of conscientiousness work hard to achieve long-term goals, follow the social rules, and feel guilty when they do not meet others’ expectations (Judge et al., 2002). Conscientiousness involves a volitional and dependability component. The will to achieve, self-motivation, and efficaciousness represent the volitional component (Le Pine et al., 2000) and significantly impact both the direction and intensity of effort people invest in tasks (Yeo and Neal, 2004). Orderliness, reliability, and cautiousness are linked to the dependability component, which represents “the tendency of people to seek order and structure in their lives, to be trustworthy and to think before act” (Le Pine et al., 2000, p. 582).

Conscientiousness represents a general work involvement tendency, resulting in satisfying work rewards (Judge et al., 2002) and, subsequently, in job satisfaction (Lapierre and Hackett, 2007). Working hard, conscientious people can perform better in their work-related roles, and job performance provides them more intrinsic and extrinsic rewards, and consequently, job satisfaction. For instance, previous studies showed that perseverance influences job satisfaction (Topino et al., 2021), people with a high level of conscientiousness being interested in long-term gain, delaying gratification, and investing effort in achieving their goals (Steel et al., 2008, 2019). Also, Perera et al. (2018) found that high conscientious teachers’ pay more attention to preparing their activities, choosing appropriate teaching strategies, managing and involving students in educational tasks, which make them experience a high level of satisfaction.

### Resistance to Change as a Mediator

Dispositional resistance to change, defined as the “people’s tendency to resist or avoid making changes, to devalue change generally, and to find change aversive across diverse contexts and types of change” (Oreg, 2003, p. 680), includes four dimensions (Oreg, 2006, 2018; Battistelli et al., 2013): cognitive rigidity (a form of stubbornness referring to people’s difficulty in changing their minds or considering alternative ideas), routine seeking (how comfortable feel people with their routines and a stable environment), emotional reactions (the feeling of anxiety imposed by changes), and short-term focus (the concern about changes’ short-term negative consequences).

Although most of the research analyzed dispositional resistance to change as an overall construct, some authors recommend looking upon each construct dimension separately to understand better their effects (Laumer et al., 2016; Kalman and Bozbayindir, 2017). Because one of these dimensions – *cognitive rigidity* – is essential in predicting how individuals perceive imposed organizational change (Laumer et al., 2016), its role has been investigated more carefully. The *cognitive rigidity* implies a low level of flexibility in ideas, views, and behaviors, with the unwillingness to consider alternative perspectives (Saksvik and Hetland, 2009; Steinmetz et al., 2011). When the need for change is imposed, people have to give up their old habits and routines and face challenges. New situations imply a different way of thinking or acting, and people may perceive them as unstructured, unfamiliar, and threatening, feeling anxious, and having difficulties adapting their behavior (Klassen and Chiu, 2010; Cullen et al., 2014). They become worried about work-related consequences (Cullen et al., 2014), about how these changes may impact their skills, qualities, or workload (Kondakci et al., 2015), which, in turn, can cause low job satisfaction. Rigid people have a high level of stubbornness and over-confidence. They prefer routines and familiar situations, the need for order and structure being both common characteristics for conscientiousness (Steinmetz et al., 2011). Hence, conscientiousness becomes a critical feature that can help individuals to work diligently and persevere when facing changes (Rafferty and Griffin, 2006) because conscientious people try to restructure the environment and make it more manageable (Steinmetz et al., 2011, p. 98). So, conscientious individuals prefer stable environments, seek routines, and are resistant to changes, having difficulties adjusting their behavior to new circumstances (Steinmetz et al., 2011). But, if they experience change, they can successfully cope with it based on clear guidelines and routines (Saksvik and Hetland, 2011). Starting from these findings, we assumed that cognitive rigidity could better explain the relationship between conscientiousness and teachers’ job satisfaction.

### Achievement Goals Orientation as a Moderator

Achievement goals are considered the “purposes that people pursue as they engage in achievement tasks” (Skaalvik and Skaalvik, 2013, p. 200). There are two types of goals: learning and performance goals. *Learning goals* are linked to the competence that people want to achieve, focusing on learning and mastery the task. When setting *performance goals*, individuals strive to be better than others, to prove their abilities relative to others (Papaioannou and Christodoulidis, 2007; Zong et al., 2018).

Although only a few studies investigated teachers’ achievement goals, the results emphasized they predominantly pursue learning goals (Papaioannou and Christodoulidis, 2007), associated with effort, planning, persistence, and self-determination to overcome the obstacles (Parker et al., 2012). Papaioannou and Christodoulidis (2007) found a positive association between learning goals and job satisfaction and no relation to performance goals. Skaalvik and Skaalvik (2013) showed that learning goals are essential for teachers’ engagement and job satisfaction, while the relationship to performance goals is positive but weak. Also, investigating the teachers’ perception of which goals are emphasized and valued at the school they work in, the authors found that learning goal structure predicts a higher level of belonging, engagement, and teachers’ job satisfaction (Skaalvik and Skaalvik, 2013). In another research, the same authors demonstrated an indirect relation between learning goals structure and teachers’ job satisfaction through self-efficacy (Skaalvik and Skaalvik, 2017).

People who pursue learning goals believe that competence can be increased by developing new skills associated with specific behavior such as planning, persistence, and hard work (Saksvik and Hetland, 2009; McCabe et al., 2013). Through the dispositional achievement orientation and dutifulness applied to the work-related tasks, conscientious people can experience success, and subsequently, job satisfaction (Perera et al., 2018). Moreover, there is a strong relationship between all facets of conscientiousness (competence, order, dutifulness, achievement striving, and deliberation) and learning goals orientation (McCabe et al., 2013). Based on the above findings, we predict that learning goals orientation will buffer the relationship between conscientiousness and teachers’ job satisfaction.

### The Present Study

Previous findings showed that teachers’ job satisfaction can be the result of the interaction between people’s characteristics and different variables from their work environment. Trying to offer a comprehensive perspective on this interaction, Lent and Brown (2006) proposed a model that integrated some of these variables (Duffy and Lent, 2009). The present study investigated two of these components: the personality traits (i.e., conscientiousness and cognitive rigidity) and teachers’ goals orientation (i.e., learning goals).

Conscientiousness, through its facets (e.g., scrupulousness and perseverance), leads to positive outcomes, and, consequently, to job satisfaction, being one of the most relevant personality dimensions associated with job satisfaction (Topino et al., 2021). Characterized by orderly, competence, and self-discipline, conscientious people prefer good routines and look for them when confronted with changes (Saksvik and Hetland, 2009). People with high levels of cognitive rigidity choose routine and familiar situations as well, which can make them less efficient in dealing with the new situation (Steinmetz et al., 2011). Previous research found positive associations between conscientiousness and cognitive rigidity, the need for order and structure being two common important characteristics with great impact on people’s ability to control situations (Steinmetz et al., 2011). Also, the majority of the conscientiousness’s facets (e.g., competence, order, dutifulness, achievement strivings, and deliberation) predict teachers’ mastery goals orientation, which impact work motivation and, finally, engagement and job satisfaction (Skaalvik and Skaalvik, 2013). Considering all the findings mentioned above, the following hypotheses were formulated:

H1. Conscientiousness will be positively related to teachers’ job satisfaction.
H2. Conscientiousness will be positively related to cognitive rigidity.
H3. Cognitive rigidity will be positively related to teachers’ job satisfaction.
H4. Cognitive rigidity will partially mediate the positive relationship between conscientiousness and teachers’ job satisfaction.
H5. Learning goals orientation will moderate the relationship between conscientiousness and teachers’ job satisfaction.

## Materials and Methods

### Participants

A total of 321 teachers were involved in this study. The convenience sample was made up of 43 males (13.4%) and 278 females (86.6%), aged between 24 and 66 (*M* = 42.70, SD = 9.72), having between 3 and 41 years of teaching experience (*M* = 18.55, SD = 9.64). They were selected from different Romanian public schools: 140 participants teach in middle school (43.6%), and 181 teach in secondary school (56.4%); 81% from the urban area, and 18.7% from the rural area. Because we were interested in their satisfaction with the teaching job, a minimum 3-year job tenure requirement in selecting teachers was used. The participants’ initial number was 336, but 15 were excluded because their job tenure was less than 3 years. Ghavifekr and Pillai (2016) showed there is a need for 2–5 years of tenure to adapt to the organizational culture and to get confirmation as a teacher.

### Procedure

One of the authors is involved in teacher training programs in the university and has a large network of collaboration with schools’ principals across the country. She contacted them, explained the purpose of this study, and asked for help to inform school teachers about the research and the possibility of getting involved in it. The principals informed their school teachers about the research and the voluntary character of their participation. Also, informed consent was obtained from all the participants included in the study. Anyone who wanted to participate in the study received a link to fill in four questionnaires online. Approximately 30 min were needed to complete these questionnaires. They were assured about the answers’ confidentiality and anonymity, and there was no reward for participation. The only exclusion criterion was the one related to the existence of at least 3 years of experience as a teacher (Ghavifekr and Pillai, 2016). All the procedures were following the ethical standards of the Institutional Research Committee, being under the 1964 Helsinki declaration and its later amendments or comparable Ethical Standards.

### Instruments

*Conscientiousness* was measured using one scale from the International Personality Item Pool–50 (IPIP-50, Goldberg, 1999) which assesses the five personality traits. The instrument was adapted to the Romanian culture by Rusu et al. (2012) and has five scales with ten items for each dimension. The participants were asked to rate statements concerning their conscientiousness on a five-point Likert scale ranging from 1 (*strongly disagree*) to 5 (*strongly agree*). An example of an item from the scale is: “I do things according to plan”. The Alpha Cronbach’s coefficient value of the scale was 0.71.

*Cognitive rigidity* was measured with 4 items scale from the Resistance to change scale (Oreg et al., 2008). The respondents were asked to rate their agreement to the statements’ content on a 6-point Likert scale (1 – *strongly disagre*e to 6 – *strongly agree*). An example of an item is: “My views are very consistent over time.” The questionnaire was already used in another Romanian research on teachers’ samples (Paloş and Gunaru, 2017). For this sample, the reliability for the cognitive rigidity was 0.74.

*Learning goals orientation* in professional development were assessed with one sub-scale from the Goal orientation scale (Attenweiler and Moore, 2006). The sub-scale of learning goals has eight items (e.g., “I enjoy challenging and difficult tasks where I learn new skills”). Participants were asked to respond on a 7-point Likert scale that ranged from 1 (*strongly disagree*) to 7 (*strongly agree*). The Alpha Cronbach’s coefficient value was 0.90.

*Teachers’ job satisfaction* was assessed with the Teaching Satisfaction Scale (Ho and Au, 2006), which measures teachers’ overall impression of their work. The questionnaire consists of five items and asks participants to respond on a 5-point Likert scale (1 – *strongly disagree* to 5 – *strongly agree*) how they feel about their job satisfaction in various ways (e.g., “I am satisfied with being a teacher,” “In most ways, being a teacher is close to my ideal”). The Alpha Cronbach’s coefficient value for the scale was 0.80.

Except for the personality questionnaire (IPIP-50), which has previously been adapted to the Romanian culture (Rusu et al., 2012), and the resistance to change which was already used in research on teachers, for the other two Romanian instruments version the standard back-translation technique was used.

### Data Analysis

After all the answers were collected, the data were analyzed based on the path analysis framework. All variables had normal distributions (Skewness and Kurtosis <1): items below three are usually acknowledged for skewness, and items below 10 for kurtosis (Kline, 2005).

To verify our hypotheses, based on the recommendation of Preacher et al. (2007), we conducted a mediation and moderation analysis, using the PROCESS macro in SPSS 22.0 (Hayes, 2013). The indirect effect was tested based on a bias-corrected bootstrapping procedure with 10,000 samples. A bootstrap confidence interval (95% CI) that does not include the “0” value signals a significant effect.

## Results

Table 1 displays descriptive statistics, reliabilities, and the correlations matrix. All the internal consistency coefficients had acceptable values, and the correlations were statistically significant. Initial support to hypotheses was evident in the correlational analysis as depicted in Table 1. To test mediation and moderation effects, Model 5 of Hayes (2013) PROCESS Macro was used in this study.

**TABLE 1:** Descriptive statistics, reliabilities, and correlation coefficients for all the variables.

Variables	*M*	SD	1	2	3	4
1. Conscientiousness	39.35	5.42	(0.71)			
2. Cognitive rigidity	12.80	2.66	0.25**	(0.74)		
3. Teachers’ job satisfaction	20.19	3.73	0.18**	0.31**	(0.80)	
4. Learning goals orientation	49.41	5.07	0.23**	0.18*	0.32**	(0.90)

**p < 0.001, *p < 0.05, N = 321; one-single tails; Cronbach’s α coefficients are presented on the main diagonal.

### Direct Effects

Based on path analysis, conscientiousness was positively associated with teachers’ job satisfaction (β = 0.08, *t* = 2.13, *p* = 0.03), supporting the first hypothesis of the study. As observed in Table 2, conscientiousness was positively related to cognitive rigidity (β = 0.12, *t* = 4.65, *p* < 0.001). Moreover, cognitive rigidity was positively related to teachers’ job satisfaction (β = 0.40, *t* = 5.24, *p* < 0.001). These results confirmed H2 and H3.

**TABLE 2:** Mediation and moderation results.

	*Outcomes*					
	*Cognitive rigidity*			*Teacher’s job satisfaction*		
*Variables*	*Coeff.*	*SE*	*p*	*Coeff.*	*SE*	*p*
Conscientiousness	0.124	0.026	<0.001	0.704	0.284	<0.01
Cognitive rigidity	–	–	–	0.338	0.074	<0.001
Learning goals orientations	–	–	–	0.675	0.214	<0.001
Conscientiousness × Learning goals orientation	–	–	–	−0.013	0.005	<0.01
*R* ^2^	0.063		<0.001	0.183		<0.001
*F*	21.70		<0.001	17.69		<0.001

*N = 321.*

### Mediation Analysis

Additionally, cognitive rigidity significantly mediated the relationship between conscientiousness and teachers’ job satisfaction (Table 2). Thus, cognitive rigidity, as mechanism to resistance to change, partially mediated the relationship between conscientiousness and teachers’ job satisfaction [indirect effect = 0.04, SE = 0.01, 95% CI (0.02, 0.08)]. These results offer support for H4.

### The Moderating Effect of Teachers’ Learning Goals Orientation

The interaction between conscientiousness and learning goals orientation is significant for teachers’ job satisfaction (β = −0.01, *t* = −2.33, *p* < 0.01). The conditional effects of conscientiousness on teachers’ job satisfaction (moderated by learning goals orientation) are presented in Table 3. The results show that the effect is significant when learning goals orientation is low [*b* = 0.083, 95% CI (0.004, 0.163)], becomes non-significant when learning goals orientation is moderate [*b* = 0.043, 95% CI (−0.029, 0.115)] and when learning goals orientation is high [*b* = −0.024, 95% CI (−0.117, 0.069)]. These results support H5.

**TABLE 3:** Conditional effects of conscientiousness, moderated by learning goals orientation.

*Values*	Learning goals orientation	*Effect*	*Bootstrap SE*	*95% Bootstrap CI*
Mean – SD	46	0.083	0.040	(0.004, 0.163)
Mean	49	0.043	0.037	(−0.029, 0.115)
Mean + SD	54	–0.024	0.047	(−0.117, 0.069)

**FIGURE 2:**
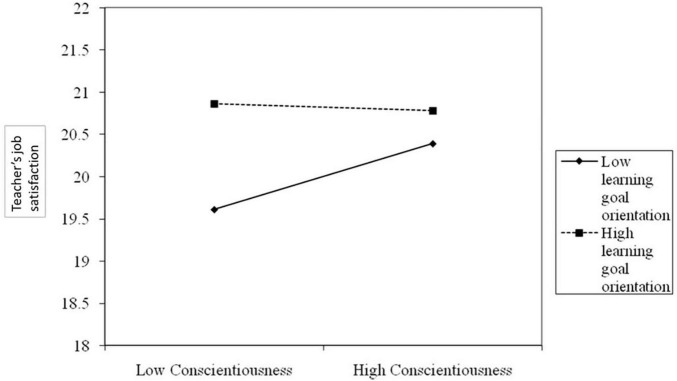
Interaction effect of conscientiousness and learning goal orientation in predicting teachers’ job satisfaction.

We plotted these significant interactions at ±1 SD from the mean of conscientiousness (see Figure 2; Aiken and West, 1991). Simple slope analysis indicated that conscientiousness significantly predicts higher teachers’ job satisfaction only when their learning goals orientation is low. As shown in the simple slope chart in Figure 2, when confronted with low learning goals orientation, teachers having a high level of conscientiousness reach a high level of job satisfaction [*b* = 0.39, *t*(321) = 8.72, *p* < 0.001].

## Discussion

The present study aimed to test the relationships between personality traits (i.e., conscientiousness and cognitive rigidity), goals orientation (i.e., learning goals), and teachers’ job satisfaction, based on the Lent and Brown (2006) model. Thus, our findings showed that cognitive rigidity, as a mechanism of dispositional resistance to change, partially mediated the relationship between conscientiousness and teachers’ job satisfaction. Learning goals orientation moderates the relationship between conscientiousness and teachers’ job satisfaction as well.

In the mediating model, teachers’ level of conscientiousness increases the likelihood of their cognitive rigidity, which, in turn, was related to a higher level of job satisfaction. Conscientiousness has a direct relation with teachers’ job satisfaction as well. Our results are in line with Topino et al. (2021) findings that reported a significant interaction between conscientiousness and job satisfaction, especially through perseverance facets which motivate them to strive for success. Also, Steel et al. (2019) found that the main driver of conscientiousness people is their willingness to achieve and to be good at their work. This can bring them recognition of their accomplishments and the feeling of job satisfaction (Organ and Lingl, 1995). A high level of conscientiousness can make people less flexible and ready to change (Saksvik and Hetland, 2009), high conscientious people being more rigid and conformist (Cohen, 2017), especially when the environment encourages these specific characteristics (George and Zhou, 2001). Teaching job involves both routines and challenges. Usually, routines are linked to administrative tasks (e.g., filling documents or statistics), and performing routine or monotonous tasks may create the feeling of comfort and a stable environment, making people with a high level of resistance to change to be satisfied with their work (Oreg, 2018). Moreover, it seems that cognitive rigidity may facilitate teachers’ performance in non-creative tasks like administrative demands which do not involve flexibility (Oreg, 2018). At the same time, challenges can refer to relationships with students and their parents, new technology and practices, and so on (Klassen and Chiu, 2010). Thus, to feel comfortable, teachers may try to find routines even in the new situations they face in their activities (Le Pine et al., 2000). When the way of thinking or acting needs to be changed, teachers may become worried about the benefits of the change, how much effort is needed, and how to adapt to the new situation. In such circumstances, the cognitive dimension (i.e., cognitive rigidity) tries to identify the change’s value, shaping the affective reactions and the commitment to the change, and, finally, teachers’ appropriate behaviors (Avidov-Ungar and Magen-Nagar, 2014).

The moderating model showed that teachers’ job satisfaction was predicted by conscientiousness only when their learning goals orientation was low. In other words, high conscientious teachers can experience job satisfaction even if they do not pursue learning goals. Although previous research indicated a positive relationship between teachers’ job satisfaction and learning goals orientation (Papaioannou and Christodoulidis, 2007), there are at least two possible explanations for our findings. Teachers who settle learning goals seek challenges, invest effort in tasks, are interested in teaching, and improve their knowledge and skills (Parker et al., 2012). They see obstacles as malleable and try to overcome them through planning, persistence, and self-management (McCabe et al., 2013), all of which are characteristics of conscientious people. So, even when the learning goals orientation is not explicit, the volition component of conscientiousness linked to achievement (i.e., hard work, persistence, and goal-orientation) influences teachers’ direction and intensity effort (Yeo and Neal, 2004). Thus, they work diligently to improve their competence and be efficient, which brings them more rewards at work and, consequently, job satisfaction (Judge et al., 2002). Also, conscientious people approach their work very seriously and are very careful and thorough (Yeo and Neal, 2004), which helps them perform better and experience success. These rewards increase their sense of accomplishment and enhance their job satisfaction (Lapierre and Hackett, 2007). The perception of their efficiency has a significant impact on job satisfaction and the relationship with students, parents, and co-workers, which is considered the primary source of job satisfaction (Gil-Flores, 2017). So, they can experience satisfaction both from their work and the relationships they have with others.

Overall, our results indicated that teachers’ job satisfaction is predicted by conscientiousness, both directly and indirectly. Conscientious teachers tend to experience satisfaction if they keep their traditional way of doing things (e.g., teaching, learning, or assessing) in a well-organized and structured manner and are not forced to change something, find alternatives, or a new perspective. People with a high cognitive rigidity are very stubborn and self-confident and, consequently, satisfied with their work results (Oreg et al., 2008). In this case, they can experience satisfaction with their job only if the change is programmed carefully, there is enough information about it, the pace of change gives them opportunities to learn how to cope with new conditions, and they are not forced to change their routines into an ambiguous environment quickly (De Simone et al., 2016).

### The Theoretical and Practical Implication

The present results emphasize the importance of conscientiousness for teachers’ job satisfaction and add new insights into social cognitive career theory, in general, and to Lent and Brown’s (2006) model, in particular. Thus, conscientiousness should be considered a coping resource for teachers, helping people work diligently and persevere when faced with change (Rafferty and Griffin, 2006). Moreover, resistance to change is another critical dimension for implementing changes in the educational environment. This contributes to understanding the relationship between conscientiousness and teachers’ job satisfaction (Laumer et al., 2016).

However, knowing how highly resistant teachers are to change, organizations can prepare them to accept and promote changes. For instance, a high level of resistance is more likely to determine negative attitudes toward change (Oreg, 2006; Laumer et al., 2016). Changes force people to search for alternatives and try to do things differently, which may cause discomfort, anxiety, insecurity, and, consequently, dissatisfaction (Kalman and Bozbayindir, 2017). So, the first step to reducing all these consequences is to offer teachers information and explain the change’s benefits. Previous findings indicated a higher cognitive rigidity level in teachers who do not get enough information related to change (Kalman and Bozbayindir, 2017). This is another reason why school management needs to be open to sharing information with their teachers (Gori and Topino, 2020) because they may resist when they “do not believe it is worth their time, effort and attention” (Yılmaz and Kılıçoğlu, 2013, p. 16).

Although some authors consider resistance to change as a stable personality trait (Oreg, 2006), some components of the resistance to change can be shaped. For example, teaching is influenced by the system of values or beliefs that drive teachers in their activity (Skaalvik and Skaalvik, 2015). Because they cannot be separated from the cognitive rigidity, school management needs to be preoccupied with these beliefs (Cohen, 2017) and agree with the implemented changes. Also, because of the frequency of change, how the change is planned, and its impact on people’s activities are significant for job satisfaction (Rafferty and Griffin, 2006), school administration should ensure that teachers receive all the support in a changing environment. Moreover, when the reforms are initiated, policymakers should cooperate with and consult all the actors involved in the educational process to explain why they are necessary and give them information about how these changes impact their work.

### Limits of the Study and Future Directions

Despite its strengths, the present study also has some limits. Thus, the sample’s unbalanced structure (more female than male teachers) can be considered a first limitation. Previous studies showed that gender is a good predictor for job satisfaction, but the results are inconsistent: some of them found that female teachers experience more job satisfaction than male teachers (Perrachione et al., 2008; Gil-Flores, 2017; Toropova et al., 2021), while others indicated that women are less satisfied with their job (Klassen and Chiu, 2010). Another limitation is the cross-sectional design that did not determine the causal relationships between all these variables. For future research, there is a need to carry out a longitudinal study to understand how, in the context of reforms, teachers’ job satisfaction is shaped by their conscientiousness and resistance to change. Also, the generalization of the results needs precautions because of at least two reasons. First, they are based on data obtained by using self-report questionnaires. Second, some surveys showed that Romanian teachers have a higher level of job satisfaction than teachers in other European countries – more than 90% of them are satisfied with the job and are confident in their abilities [Teaching and Learning International Survey (TALIS), 2013].

## Conclusion

Our results demonstrated new insights regarding the relationships between psychological variables (i.e., conscientiousness, resistance to change, and achievement goals orientation) and teachers’ job satisfaction. Thus, cognitive rigidity represents a crucial mediating link between conscientiousness and teachers’ job satisfaction. Moreover, the moderating role of learning goals orientation manifests in the relation between conscientiousness and job satisfaction. These results also emphasize how useful it can be in the educational environment to prepare for the changes. School management can offer teachers information and explain the change’s benefits to prevent individual resistance to change and decrease satisfaction related to their work.

## Data Availability Statement

The raw data supporting the conclusions of this article will be made available by the authors, without undue reservation.

## Ethics Statement

Ethical review and approval was not required for the study on human participants in accordance with the Local Legislation and Institutional Requirements. The participants provided their written informed consent to participate in this study.

## Author Contributions

RP has chosen the topic and contributed to the design, methodology, writing, and supervision of the present manuscript. DV contributed to the design, methodology, writing, and supervision of the present manuscript. MC coordinated the collection of the data and contributed to the design and writing of the manuscript. All authors contributed to the article and approved the submitted version.

## Conflict of Interest

The authors declare that the research was conducted in the absence of any commercial or financial relationships that could be construed as a potential conflict of interest.

## Publisher’s Note

All claims expressed in this article are solely those of the authors and do not necessarily represent those of their affiliated organizations, or those of the publisher, the editors and the reviewers. Any product that may be evaluated in this article, or claim that may be made by its manufacturer, is not guaranteed or endorsed by the publisher.
